# Uracil within DNA: an actor of antiviral immunity

**DOI:** 10.1186/1742-4690-5-45

**Published:** 2008-06-05

**Authors:** Joséphine Sire, Gilles Quérat, Cécile Esnault, Stéphane Priet

**Affiliations:** 1 UMR IRD-190, Emergence des Pathologies Virales, Faculté de Médecine, 27 Bd Jean Moulin, 13005 Marseille, France; 2 Unité des Rétrovirus Endogènes et Eléments Rétroïdes des Eucaryotes Supérieurs, UMR 8122 CNRS, Institut Gustave Roussy, 94805 Villejuif, France; 3 Architecture et Fonction des Macromolécules Biologiques, CNRS UMR 6098, ESIL case 925, 13288 Marseille Cedex 9, France

## Abstract

Uracil is a natural base of RNA but may appear in DNA through two different pathways including cytosine deamination or misincorporation of deoxyuridine 5'-triphosphate nucleotide (dUTP) during DNA replication and constitutes one of the most frequent DNA lesions. In cellular organisms, such lesions are faithfully cleared out through several universal DNA repair mechanisms, thus preventing genome injury. However, several recent studies have brought some pieces of evidence that introduction of uracil bases in viral genomic DNA intermediates during genome replication might be a way of innate immune defence against some viruses. As part of countermeasures, numerous viruses have developed powerful strategies to prevent emergence of uracilated viral genomes and/or to eliminate uracils already incorporated into DNA. This review will present the current knowledge about the cellular and viral countermeasures against uracils in DNA and the implications of these uracils as weapons against viruses.

## Background

Uracils in DNA may arise either from incorporation of dUTP in place of thymidine 5'-triphosphate (dTTP) or from the generation of uracils in DNA consecutive to spontaneous or enzymatic deaminations of cytosines which, if unrepaired, will lead to non-mutagenic U:A or mutagenic U:G mispairs, respectively. Although U:A mispairs resulting from excess of cellular dUTP pool levels are not mutagenic *per se*, they elicit a cycle of dUMP incorporation into DNA followed by the removal of uracil base by cellular uracil DNA glycosylases (UNG) and reincorporation of dUMP during the synthesis phase. The end point of this process is the appearance of strand breaks and the loss of DNA integrity. In nonproliferating cells such as macrophages, quiescent lymphocytes or neurons the intracellular deoxynucleotide pool is low and imbalanced, with high levels of dUTP, due to the limited expression of the deoxyuridine 5'-triphosphatase nucleotide hydrolase (dUTPase) that otherwise controls the dUTP/dTTP ratio. Consequently, viruses that replicate in this adverse cellular context have a high probability to incorporate dUTP in their genome during viral replication. They have thus acquired strategies consisting in concentrating dUTPase or UNG activities in close proximity to their replication machinery. Most often they have done so by encoding themselves viral dUTPase and/or UNG in order to compensate for the low levels of these cellular enzymes. In the following we will focus on the different ways by which uracils are introduced into cellular and viral DNA and on the resulting biological consequences when uracils remain unrepaired, with a special attention to HIV-1 lentivirus. HIV-1 replicates in nondividing cells but does not encode dUTPase nor UNG. However, HIV-1 fights the detrimental uracilation of its genome induced by members of the APOBEC family, which are cytosine deaminases able to convert cytosine to uracil residues, through the Vif protein. Vif impedes the packaging of APOBEC members avoiding excessive G-to-A hypermutations within viral genome. The role in virus life cycle of the host-derived UNG (UNG2) enzyme that is packaged into HIV-1 virions will be discussed.

### Uracils in cellular or viral DNA may derive from different sources

The common RNA base uracil (U) that is substituted by thymine (T) in DNA is able to naturally pair with adenine (A) but can also mispair with guanine (G). The U:A pair in DNA results from the incorporation of dUTP by polymerases and constitutes a non-mutagenic event *per se *that can nonetheless alters promoters functions [1]. However, U:A pair may be a cytotoxic lesion or even become a mutagenic event when chromosomal abasic sites (AP-sites) are generated after the removal of uracils by cellular repair mechanisms [2]. The U:G mispair is a non-blocking DNA replication lesion and occurs after the deamination of a cytosine to uracil. This lesion is mutagenic, leading to a G-to-A transition mutation in one of the two daughter strands after DNA replication.

**Figure 1:**
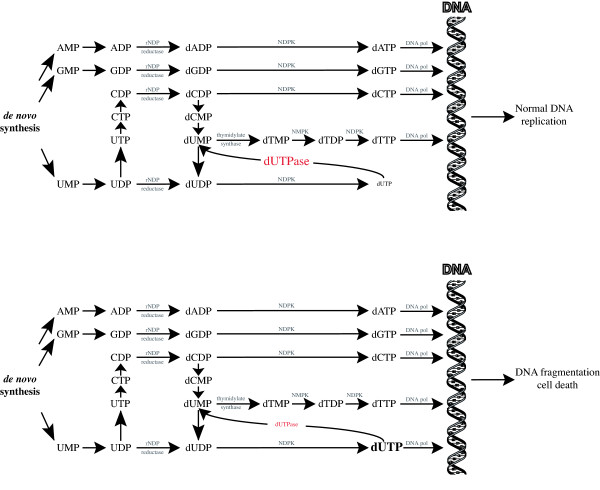
Biosynthesis pathways of ribonucleotides and deoxyribonucleotides in mammalian cells and the possible consequence of the misincorporation and repair of uracil residues in DNA. *De novo *synthesis of AMP, CMP, GMP and UMP ribonucleotides allows the formation of dATP, dCTP, dGTP, dTTP and dUTP deoxyribonucleotides, which can be readily incorporated in DNA by cellular DNA polymerases. Note that dTTP derives from dUTP hydrolysis. Abbreviations: A, adenine; C, cytosine; G, guanine; T, thymine; U, uracil; MP, monophosphate; DP, diphosphate; TP, triphosphate; rNDP, ribonucleotide diphosphate; NMPK, nucleotide monophosphate kinase; NDPK, nucleotide diphosphate kinase.

The incorporation of dUTP into DNA during replication has been estimated to be up to 10^4 ^uracil residues in human genome per day [3] and represents the major source of uracils in DNA [4]. In eukaryotic cells, dUTP is synthesized from the phosphorylation of dUDP arising either from UDP under the action of the ribonucleoside diphosphate (rNDP) reductase or from the phosphorylation of dUMP, which is an essential intermediate for the synthesis of the intracellular dTTP pool and therefore constitutes a permanent source of dUTP (Fig. 1). DNA polymerases from eukaryotes, prokaryotes and viruses are not able to discriminate dUTP from dTTP. Thus the incorporation of dUTP directly depends on its intracellular concentration. Under physiological conditions, the concentration of dUTP and dTTP in the cell have been estimated to be ~0.2 μM and 37 ± 30 μM, respectively [5] meaning that the normal intracellular dUTP/dTTP ratio is below or close to 1%. However, some cell types such as HT29 cell line, primary spleen cells, macrophages or quiescent lymphocytes display significantly higher dUTP levels that can even exceed those of dTTP [6-8].

The deamination of cytosine residues to uracil residues in DNA can arise either from a spontaneous (non-enzymatic) or an enzymatic process. Spontaneous deamination is a frequent event that has been estimated by chemical measurements and genetic assays to occur between 70 to 200 times per cell per day [9]. In addition to cytosine deaminases, the mammalian genome encodes two distinct enzymes able to convert cytosine to uracil, namely the (cytosine-5)-methyltransferase and the APOBEC cytidine deaminase. The (cytosine-5)-methyltransferase, is in charge of the conversion of cytosines within CpG islets to 5-methylcytosines. In mammalian cells, 5-methylcytosines represent about 2 to 7% of cytosines and constitute a regulatory system for transcription and can confer epigenetic informations [10]. The conversion starts with the formation of a covalent bond between the enzyme and the cytosine, leading to a transient dihydropyrimidine intermediate product that is quickly subjected to spontaneous deamination. The enzyme next catalyzes the transfer of a methyl group to the cytosine. This latter reaction uses the S-adenosylmethionine (SAM) molecule as a methyl donor. Thus, a cytosine deamination to uracil may occur in the case of the abortive catalysis by (cytosine-5)-methyltransferase [11] or in the presence of a low cellular concentration of SAM [12].

**Figure 2:**
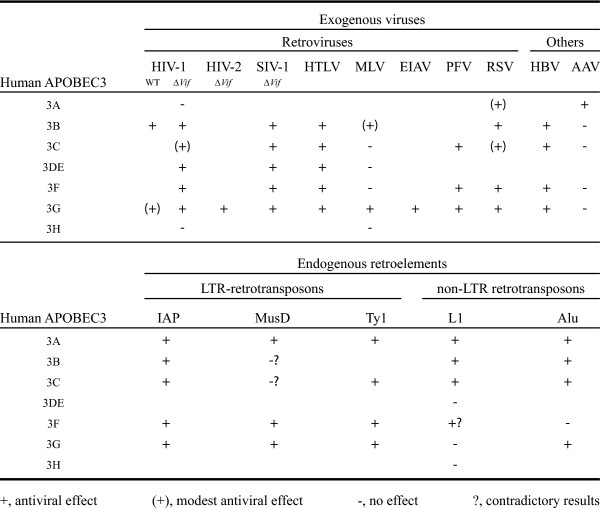
APOBEC3 family members and their associated roles in exogenous viruses and endogenous retroelements restriction. Data are compiled from [27, 77, 87, 90, 126-140].

The APOBEC cytidine deaminase family members are able to deaminate cytosines within DNA and/or RNA molecules. The first member of this family, APOBEC1 (apolipoprotein B mRNA editing catalytic subunit 1), has been identified as the enzyme responsible for the tissue-specific deamination of the C^6666 ^of the apolipoprotein B mRNA, leading to a premature stop codon and the expression of a truncated form of the apolipoprotein B lipid-transport protein in gastrointestinal tissues [13,14]. The APOBEC1 protein acts exclusively as a RNA-editing enzyme in the small intestine (where it is exclusively expressed) but can, however, deaminate cytosines present in chromosomal DNA of living bacteria [15,16]. These results drew attention to the possibility that APOBEC proteins could deaminate either RNA or DNA under different cellular conditions [15,16]. Other members of the APOBEC cytidine deaminase family, including AID (activation-induced cytidine deaminase), APOBEC2, the APOBEC3 sub-family and APOBEC4, have next been discovered and the biological function of several of them has been studied. At this time, no function has yet been attributed to APOBEC2 and APOBEC4 proteins. The AID protein, whose expression is restricted to activated mature B cells, has been identified as a key factor of antibody diversification [17]. AID is required to deaminate specifically some cytosines in ssDNA of variable and switch regions of the Ig gene locus, allowing somatic hypermutation (SHM) and the class-switch recombination (CSR) processes that are needed to generate antibody diversity in response to antigens [18-21]. The APOBEC3 sub-family has been discovered when human APOBEC3G (hA3G) was reported as a host cell restriction factor for HIV replication [22]. Subsequently, it has been reported that seven APOBEC3 proteins, so-called APOBEC3A, 3B, 3C, 3DE (the 3D and 3E genes encode the N- and C-terminal domains of the 3DE protein, respectively), 3F, 3G and 3H, are encoded by the human genome [23,24]. These APOBEC3 proteins, with the exception of APOBEC3H, have been shown to exhibit antiviral effects against a variety of viruses, including numerous retroviruses such as HIV, SIV, MLV, HTLV and foamy viruses, hepatitis B virus and adeno-associated virus (AAV) (reviewed in [25]) (Fig. 2). The absence of antiviral effect of human APOBEC3H, in contrast to its Old World monkey (OWM) counterpart, may be explained by a poor expression [26]. The antiviral effect displayed by other human APOBEC3 proteins, with the exception of APOBEC3A, was associated with numerous cytosine deaminations (known as "editing") within the viral cDNA leading to lethal G-to-A mutations (reviewed in [25]). Indeed, APOBEC3A has been found to exert antiviral effects without cytosine deaminations although recent reports showed that it was capable of editing *in vitro *on single-stranded DNA [27] and on the cDNA of the avian alpharetrovirus RSV thereby inhibiting its infectivity [28]. Studies of deaminase-defective APOBEC3 mutants have shown that APOBEC3G and APOBEC3F contain antiviral determinants that can act independently of the editing process [29,30]. However, a recent study reported that this previously described antiviral effect of the deaminase-defective APOBEC3G mutants was negligible as compared to the wild-type protein when equal amounts of these proteins were packaged into viral particles [31]. Beside the antiviral function of APOBEC3 proteins against exogenous viruses, some inhibitory effects have been reported on intracellular targets like the IAP, MusD or Ty1 Long Terminal Repeat (LTR)-retrotransposons and LINE-1 or *Alu *non-LTR retrotransposons through a general deaminase-dependent mechanism (again the with exception of APOBEC3A) [25]. Accordingly, several studies support the notion that one of the cellular functions of APOBEC3 proteins could be to prevent the propagation of mobile elements in their host genomes. In a general way, uracils coming from the action of the AID or the APOBEC3 proteins appear to be central actors in the adaptive or innate immune response, respectively.

### Genomic uracils are highly controlled in cells

Eukaryotic and prokaryotic cells have evolved in setting up two mechanisms to impede the presence of uracils in DNA, bringing to light the highly deleterious effects of genomic uracils if unrepaired. The first mechanism in place prevents the incorporation of dUTP by acting directly on the intracellular pool of dUTP through the action of dUTPase, while the second is responsible for the excision of uracil once present in DNA through an universal DNA repair process called base excision repair (BER) and the use of enzymes known as uracil-DNA glycosylases (UNGs).

The dUTPase is a ubiquitous enzyme that is well conserved in all organisms. This protein maintains a low level of intracellular dUTP by converting dUTP to dUMP and inorganic pyrophosphate and also allows the biosynthesis of nucleotides derived from thymidine [32] (Fig. 1). The human *dUTPase *gene encodes, through alternative splicing, two isoforms that localize to either the mitochondrion or nucleus [33]. The expression of the nuclear form is cell-cycle regulated with a high expression in the S phase of dividing cells that contrasts to a nearly undetectable expression in differentiated and non-dividing cells [34]. Although no human dUTPase deficiency has been observed, the absence of the dUTPase activity in prokaryotes and *S. cerevisiae *has demonstrated its necessity for cell viability. In addition, partial deficiency leads to enhanced frequency of spontaneous mutations, recombinations and DNA fragmentation [35-39].

The BER process is one of the cellular DNA repair mechanisms responsible for correcting most of common forms of DNA damage including the removing of genomic uracils. The DNA repair process has been extensively subjected to reviews [40] and will be shortly introduced here. It involves the recognition and the excision of an inappropriate base by a specific DNA glycosylase leading to an abasic site (AP-site) that is further cleaved on its 5' side by an apurinic/apyrimidinic (AP)-endonuclease APE, leaving a free 3'-OH end and a 5'-deoxyribose phosphate (dRP) group. The dRP group is then incised on its 3' side via the lyase activity (dRPase) of the DNA polymerase β (pol β) for the short patch repair pathway while a short oligonucleotide is cleaved by the flap endonuclease 1 (FEN1) for the long patch repair pathway. Finally, the resulting gap is then filled in by pol β and/or pol δ/ε and sealed by the DNA ligase I or III. The DNA glycosylases responsible for the excision of uracils are highly conserved enzymes expressed in mammals, bacteria, yeast, or herpes- and poxviruses. In humans, several enzymes with UNG activity have been described, namely TDG, MBD4, SMUG1, UNG1 and UNG2 [41]. The UNG2 and SMUG1 enzymes have been reported as the major enzymes proficient for removing deaminated cytosines although SMUG1 is thought to act as a backup of UNG2 [42-44]. UNG2 has been also reported as the unique enzyme able to perform the excision of uracil from dUTP misincorporation [42,43]. The UNG1 and UNG2 enzymes are mitochondrial and nuclear isoforms, respectively, generated by alternative splicing of the human *ung *gene [45]. Like the dUTPase, the expression of the nuclear UNG2 form depends on the cell cycle with high levels in the S phase of dividing cells, and barely detectable levels in differentiated and non-dividing cells [34]. In contrast to UNG2 that accumulates in replication foci [42,43], SMUG1 is only expressed in nucleoli where it may have a specialized role [44]. UNG-deficiency in mice and in humans leads to increased accumulation of genomic uracils, confirming its primary role in the removal of uracil from DNA [46-48].

Although UNG2 is considered to display an antimutagenic function, it seems to have an essential role in the antibody diversification process. The vast repertoire of antibody molecules, which is essential to detect and fight pathogens, is generated thanks to profound genomic changes at the Ig locus in B cells. This process occurs through numerous somatic hypermutations (SHM) that lead to the affinity maturation of antibodies, and through class-switch recombination (CSR) allowing these high affinity antibodies to gain some effector functions and to be disseminated across the body. These mechanisms are initiated from the targeted deamination of cytosines triggered by AID within the Ig locus [18-21]. The AID-generated uracils lesions are then recognized and excised by UNG2 enzyme. Replication across the resulting AP-sites by REV1 and other translesion polymerases results in the generation of transition mutations at C:G pairs [49,50]. Moreover, some mutations at A:T pairs can also be observed during the SHM process but the molecular mechanisms involved remain to be fully understood. The MSH2-MSH6 mismatch repair proteins could however recognize U:G mispairs and could be required to generate A:T mutations [51-54]. A recent study has indicated that the MSH2-MSH6 heterodimer could prevent the error-free BER commonly initiated by UNG2 and that UNG2 could recruit pol η, which appears to be the sole contributor of A:T mutations [55]. Thus, UNG2 plays a key role in SHM as well as CSR processes. Indeed, UNG2-deficient humans cannot ensure CSR and therefore have elevated IgM amounts and dramatically lowered IgG, IgA and IgE levels [46]. However, the role of the uracil excision activity of UNG2 in this process seems not clear and warrants further studies. Altogether, these data highlight the crucial role of UNG2 in the adaptative immunity against numerous pathogens, like bacteria or viruses.

### Numerous viruses have evolved strategies to counteract uracils

Viruses belonging to the *Herpesviridae*, *Poxviridae *and *Retroviridae *families have evolved in encoding their own dUTPase and/or UNG proteins, supporting the idea that these viruses are sensitive to the presence of uracil residues in their genome.

The *Herpesviridae *family contains members that replicate their dsDNA genome in the nucleus of a variety of cell types, including some non-dividing cells, such as neurons. The viral dUTPase and UNG of alpha herpesviruses, like herpes simplex virus (HSV) or varicella zoster virus (VZV), are not essential in proliferating cells [56-58] but become critical in non-dividing cells or in an *in vivo *murine system [59,60]. Beta herpesviruses, like cytomegalovirus (CMV), do not encode a functional dUTPase [61] but express their own UNG that appears to be required in non-dividing cells to compensate for the very low level of cellular UNG2 [62,63]. The gamma herpesviruses, like Epstein-Barr viruses (EBV), Kaposi's sarcoma-associated herpesviruses (HHV-8) or murine γ-herpesvirus-68 (MHV-68), also encode a functional UNG and dUTPase [64,65]. The EBV-encoded UNG was shown to ensure the fidelity of viral DNA replication and to promote efficient production of viral DNA [64] and the EBV-encoded dUTPase expressed by MHV-68 was found to play an important role in acute infection in the lung [66].

The members of the *Poxviridae *family, such as vaccinia virus, replicate their dsDNA exclusively in the cytoplasm of infected cells. Thereby, due to the total absence of cytosolic UNG activity, the inactivation of the virally-encoded UNG leads to an impaired viral replication even in proliferating cells [67,68]. These data highlight that uracils in the genome of DNA viruses can impair their replication and depict the necessity for these viruses to encode their own dUTPase and/or UNG enzymes to compensate for the lack of cellular counterpart during their life cycle either in non-dividing cells or in the cytoplasmic compartment.

Several members of the *Retroviridae *family, such as β-retroviruses including Mazon-Pfizer monkey virus (MPMV) or murine mammary tumor virus (MMTV), nonprimate lentiviruses including the ungulate lentiviruses maedi-visna virus (MVV) of sheep, the caprine arthritis-encephalitis virus (CAEV), the equine infectious anemia virus (EIAV), the feline immunodeficiency virus (FIV) and the rabbit endogenous lentivirus type K (RELIK), and even many endogenous retroviruses including HERV-L, MERV-L and HERV-K, also encode their own dUTPase protein. Interestingly, the dUTPase domain of these retroviruses can be differently located in their genome, either upstream of protease, or in-between reverse transcriptase and integrase, or downstream of integrase, indicating that retroviruses have captured a *dUTPase *gene during independent occasions. This suggests that the need of the dUTP hydrolysis activity is crucial to confer a selective advantage for retroviral replication. The virally-encoded dUTPase protein is expressed as a part of the Gag-pol polyprotein precursor and is expected to be encapsidated into viral particles in order to be in close spatial proximity of the reverse transcriptase [69]. Although the role of β-retroviral or endogenous retroviral dUTPases still remains to be fully determined, the study of the dUTPase of nonprimate lentiviruses revealed that it is required for efficient replication only in non-dividing cells, [70-74]. It is noteworthy that other retroviruses members, such as murine leukemia virus (MLV) related viruses or avian leukemia virus (ALV) related viruses, do not encode dUTPase. One can notice that they replicate their genome only in actively dividing cells, which may explain why they do not need to encode dUTPase.

Collectively, these data show that (i) although not mutagenic *per se*, uracils in viral genomes coming from dUTP misincorporation alter viral replication, (ii) high dUTP levels are present in the nucleus of non-dividing cells or in the cytoplasm of proliferating cells that express low levels of dUTPase and UNG enzymes, and finally (iii) uracils in viral DNA genome have to be highly controlled to allow an efficient viral replication.

### APOBEC3 proteins as a part of the innate anti-HIV-1 immunity and viral countermeasures

Since Sheehy *et al*. discovered that the human endogenous APOBEC3G protein acts as a restriction factor to inhibit HIV-1 replication [22], the question of innate immunity against viruses has gained much attention. From that time, several other APOBEC3 proteins, namely APOBEC3F [75,76], -3B [77,78] and -3DE [79] have been shown to exert antiviral effects against HIV-1. This restriction effect requires the packaging of APOBEC3 proteins into virions budding from HIV-1 infected cells. Studies on the incorporation of these antiviral factors into viral particles brought conflicting evidences leading to propose that either a specific interaction with the nucleocapsid (NC) domain of Gag polyprotein [80-82], or an association with viral or cellular RNA [83-86] were required for the encapsidation. Recently, studies with highly divergent Gag polyproteins [87] or RNase A treatment [83,84,88] brought more evidence to the second model where APOBEC3 packaging involves an RNA intermediate. Following the entry of APOBEC3G-containing viral particles in target cells, virion-associated APOBEC3 proteins trigger extensive cytosine deaminations to uracils in neosynthesized viral minus-strand DNA [89-92]. This process occurs through a target site specificity depending on the nature of the APOBEC3 protein. Thus, APOBEC3G prefers to deaminate the dinucleotide CC (underline marks the target) on minus-strand cDNA [89-92], while APOBEC3F or -3B favors a TC context [77] and APOBEC3DE selects a GC context [79]. This event takes place next to the degradation of the RNA template by the RNase H activity of RT because (i) the APOBEC3G proteins act only on single-stranded DNA [93] and (ii) the APOBEC3G activity is inhibited by viral genomic RNA [94]. The deamination of cytosine by APOBEC3 proteins leads to lethal G-to-A hypermutations in viral plus-strand cDNA [89-92,95]. However, in several *in vitro *experimental assays, some APOBEC3 family members are depicted as able to restrict some retroviruses, retroelements or other viruses through mechanisms independent of DNA deaminations (reviewed in [25]) (Fig. 2). In the case of APOBEC3G, it has been reported that it exhibits both deaminase-dependent and deaminase-independent antiviral activities. The deaminase-independent activity could be due to directly inhibiting the strand transfer or the processivity of reverse transcriptase [96,97].

To counteract the editing process and to protect its genome from G-to-A hypermutations, HIV-1 evolved in encoding the Vif protein that exhibits the property to bind APOBEC3G and to induce its proteasomal degradation, thus depleting APOBEC3G at the site of virion budding [98-100]. The degradation of APOBEC3G requires the formation of a Vif-Cul5-elongins B and C-Rbx1 complex, which allows the polyubiquitination of APOBEC3G. It has also been reported that Vif could interfere with APOBEC3G translation [101] or directly with its packaging [102]. Interestingly, the Vif-mediated degradation appears to be more potent on APOBEC3G than on APOBEC3F or -3DE and has not been observed for APOBEC3B [98-100]. In addition, the aspartate residue at position 128 of APOBEC3G from different species has been reported to drive the interaction with Vif and thus to govern species specificity [102-106]. Indeed, HIV-1 Vif protein was efficient to bind to and inhibit the packaging of human APOBEC3G but not that of the simian (African green monkey or rhesus monkey) or murine counterparts [102,107].

Collectively, these data show that cells take advantage of uracil susceptibility displayed by numerous viruses to inhibit their propagation through the expression of the APOBEC3 family members. Unfortunately, HIV-1 illustrates the high capacity of viruses to evolve rapidly and to develop mechanisms to thwart this innate antiviral immunity.

### Primate HIV-1 lentivirus controls uracils within viral DNA

Lentiviruses are divided into nonprimate and primate subgroups and share common characteristics, such as the ability to infect non-dividing cells and to replicate their genome in the cytoplasm of infected cells. In this cellular context, nonprimate lentiviruses control the deleterious uracilation of their genome through the expression of a virally-encoded dUTPase. The analysis of primate lentivirus genomes reveals that they do not encode dUTPase nor UNG-like enzymes. Using a yeast two-hybrid screen, cellular UNG2 was shown to associate with the accessory HIV-1 Vpr protein [108] and to be incorporated into HIV-1 viral particles [109-114]. Although Vpr was initially suspected to play a role in the packaging of UNG2 [110,111], it was later demonstrated by three other groups that UNG2 packaging was independent of the presence of Vpr in viral particles [109,112,113,115,116]. Indeed, the integrase (IN) domain in the context of the HIV-1 Gag-Pol precursor was identified as the sole determinant to target UNG2 into virions and we have demonstrated that the leucine 172 residue of IN was critical for UNG2 encapsidation [109,115]. The UNG2 packaging was specific to HIV-1, as neither HIV-2 nor simian immunodeficiency virus (SIV) packaged UNG2 [116]. It remains to be determined whether the latter package another cellular UNG or whether they manage to circumvent the uracilation of their genomes through other means.

Although it has been shown that Vpr could participate in the fidelity of reverse transcriptase [110], the role of the Vpr-UNG2 association remains to be evaluated. Interestingly, recent results showed that the expression of Vpr in B-cells had a dominant-negative effect on the class switch recombination (CSR) events [117]. This effect appeared to be dependent on the interaction of Vpr with the WxxF motif contained in UNG2 suggesting that Vpr could compete with some endogenous Vpr-like factors important for UNG function in CSR. Indeed, the CSR is initiated by AID and the AID-induced uracils are removed by a UNG2- or a MSH2-dependent mechanism [118], leading to a not fully characterized dsDNA cleavage step required for CSR (reviewed in [119]). By analogy, it is possible that the Vpr-UNG2 complex may play a role in inhibiting dsDNA cleavages subsequent to the UNG2-induced removal of uracils in viral cDNA. This assumption may explain why the infectivity of HIV-1 virions that contain small amounts of APOBEC-induced uracils was increased by Vpr [113] and why Vpr seems to be important for viral replication in non-dividing cells [111]. Future experiments demonstrating a direct implication of the Vpr-UNG complex in the protection of dsDNA from cleavage are awaited. Another point that deserves future investigations concerns the fact that Vpr could potentially target hAPOBEC3G, which contains in its sequence two WxxF motifs. If such a Vpr-hAPOBEC3G interaction exists, it could assign an additional role of Vpr as a back-up of Vif.

The role of the virion-associated UNG2 in the viral life cycle was evaluated by our laboratory through the specific depletion of UNG2 by RNA interference-mediated knock-down and through the inhibition of UNG2 catalytic activity by the expression of the uracil-DNA glycosylase inhibitor (Ugi) in virus producing cells [120]. It was found that HIV-1 viruses produced from dividing as well as nondividing UNG2-deficient cells were noninfectious, demonstrating that UNG2 within the viral particle is essential for infectivity. This also demonstrated that the depletion of UNG2 in viral particles could not be compensated by endogenous UNG2 present in target cells, suggesting that the spatial proximity of UNG2 and the replication enzymes in the viral nucleocapsid is important. In addition, the targeting of the CAEV dUTPase into UNG2-deficient viral particles was able to rescue the viral infectivity, pointing out that dUTP misincorporation could be at the origin of a lethal reverse transcription process. Indeed, HIV-1 lysates contained all necessary factors to repair uracils originating from dUTP misincorporation during the reverse transcription process [120,121]. These data pointed out that, to prevent dUTP misincorporations, HIV-1 is endowed with an original uracil BER pathway that is initiated by UNG2 and associated with a previously unknown dNTP-dependent AP-endonuclease activity of reverse transcriptase (RT) [120]. Although others [110] and we [121] failed to identify the human AP-endonuclease APE1 within HIV-1 particles, a recent report indicated that APE1 molecules could be detected in viral particles [114]. The presence of APE1 within viral particles could participate in association with UNG2 and RT to the repair of dUTP misincorporations and could thus have a redundant function in HIV-1 particles. Meanwhile, the main role of APE1 within viral particles seems rather to be the degradation of APOBEC3G-edited nascent HIV-1 DNA after the excision of uracils by UNG2 [114], suggesting a specialized function of UNG2 and APE1 hijacked by the cell to amplify the anti-HIV-1 effect of APOBEC3G in the absence of Vif. It should be mentioned that the degradation of newly synthesized transcripts cannot be performed by the dNTP-dependent AP-endonuclease activity of RT because (i) the RT can cleave UNG2-induced AP-sites only during the reverse transcription step, i. e. when a template is available (be it RNA or DNA) [120,122], (ii) the substrate for APOBEC3G is the ssDNA [93,95] and, (iii) the degradation of the viral RNA template by the RNase H activity of RT, which inhibits the APOBEC3G activity [94], is a prerequisite for the cytosine deamination process [93,95].

Our data demonstrating that UNG2 was necessary for viral infectivity are in sharp contrast with data from two other groups [112,114] showing that UNG2 was somewhat dispensable for HIV-1 replication. Kaiser and Emerman reported that, although UNG2 was efficiently encapsidated into HIV-1 virions, it played no role in infectivity, neither for wild type (WT) viruses nor for Vif-minus viruses in the presence of APOBEC3G [112]. In contrast, Yang's group has reported that UNG2 encapsidation from producer cells, while dispensable for replication of WT viruses, was involved in the rescue of Vif-minus HIV-1 in the presence of APOBEC3G [114]. Although all groups target UNG2 inhibition or depletion (by the use of Ugi as an inhibitor of UNG2 activity, UNG2 specific siRNA or UNG2-/- mutants cells), it is difficult to reconcile these conflicting data given that methodologies differ. However, two drawbacks have not been addressed. First, the quasi-irreversibility of the binding of Ugi to UNG has been demonstrated for pure *E. Coli *UDG [123,124] and not for human UNG2 either pure or already engaged in its interaction with HIV-1 integrase (IN). Therefore it remains possible that the incoming Ugi associated to the virally-encapsidated UNG2 may well dissociate by competition with the vast excess of free UNG2 in the recipient cells, leading to a functional UNG2-positive entering replication complex. Second, the EBV transformed B cell lines from *UNG*^-/- ^patients may well express high levels of EBV-encoded dUTPase and/or UNG. Thus, the possibility exists that EBV-encoded UNG could be packaged into HIV-1 particles and compensates for the lack of human UNG2 in the *UNG*^-/- ^cell line. This hypothesis might be verified by analyzing the EBV-transformed cell line for the expression of EBV-encoded UNG and its packaging to HIV-1. In addition, the expression of EBV-encoded dUTPase will be sufficient to abrogate the cytoplasmic dUTP pool levels. Therefore, the best approach to definitely conclude on the role of UNG2 in HIV-1 infectivity would be the use of primary T lymphocyte or macrophages, instead of EBV-transformed B cell lines, derived from *UNG*^-/- ^patients and would thus avoid some manipulations of cells including overexpression of Ugi or RNA interference. In addition, one can try to make his mind about the role of UNG2 in the HIV-1 life cycle by some considerations of evolutionary perspectives. Yang's data point to a positive role of UNG2 packaging only when Vif is deficient, but are neutral for a WT virus. It is difficult to pinpoint any evolutionary forces, which could select for UNG2 packaging in such a case. The Kaiser and Emerman data are consistent with an accidental packaging of UNG2, an epiphenomenon not related with any evolutionary advantage. Such hypothesis would explain the lack of UNG2 packaging into HIV-2 and SIV virions, two closely related cousins of HIV-1, although no one has still looked at whether other cellular UNGs might be encapsidated. In contradiction with that hypothesis is the presence in all other non-primate lentiviruses of an enzymatic activity, so-called dUTPase, in charge of preventing uracilation of their genome. Given that all lentiviruses infect non-dividing macrophagic and dendritic cells as their main and common target cells, it is tempting to speculate that they acquired means to use enzymatic activities enhancing their replication in the adverse context of nondividing cells. This hypothesis is conforted by the fact that an ancestral dUTPase gene was identified in the HIV-1 envelop gene [125].

Taken together, these data sustained the notion that the presence of uracils in viral reverse transcripts is a constant threat for HIV-1 replication. The virus consequently evolved in developing strategies including both the expression of the virally-encoded Vif protein to inhibit the packaging of host-derived cytosine deaminase APOBEC proteins and the packaging of host-encoded uracil DNA glycosylase UNG2 to initiate uracil repair of misincorporated dUTP.

### Concluding remarks

To date, considerable progress has been made in understanding the interactions between superior organisms and their pathogens. The discovery of the APOBEC family members has highlighted a central role for uracils as a barrier against infectious agents, firstly as an actor of antibody diversification in adaptative immunity, and secondly as a potent antiviral *per se*. This latter feature has been particularly well documented in the case of infection by HIV-1 and revealed how this virus faces up to the uracilation of its genome. During the natural course of HIV-1 infection, the Vif expression level in infected cells seems to be sufficient to avoid extensive APOBEC3G-mediated cytosine deamination of the viral genome that otherwise will lead to error catastrophe. It remains possible however that some APOBEC3G proteins escape the Vif-mediated degradation. This in turn could help in the emergence of G-to-A mutations enhancing drug resistance and immune escape processes of HIV-1. Likewise, UNG2 encapsidation level within viral particles seems to be sufficient to prevent the dUTP incorporation in viral reverse transcripts. However, nothing is known about the efficiency of HIV-1 replication when infected cells exhibit high levels of intracellular dUTP. Therefore, it may be interesting to test whether enhancing artificially the dUTP pool levels in HIV-1 infected cells may represent an alternative approach to antiviral therapy. This could be done by inhibition of thymidylate synthase or dUTPase. The development of drugs against these enzymatic activities may deserve to be evaluated as a viable therapeutic approach.

## List of abbreviations

dUTP: deoxyuridine 5'-triphosphate nucleotide; AID: activation-induced cytidine deaminase; APOBEC: apolipoprotein B mRNA editing catalytic polypeptide; UNG: uracil DNA glycosylase; dUTPase: deoxyuridine 5'-triphosphatase nucleotide hydrolase; BER: base excision repair; SHM: somatic hypermutation; CSR: class-switch recombination; HIV-1: immunodeficiency virus type-1; Vif: viral infectivity factor.

## Competing interests

The authors declare that they have no competing interests.

## Authors' contributions

JS, GQ, CE and SP participating equally in revising the intellectual content and drafting of the manuscript. Author(s) read and approved the final manuscript.
